# Update on extranodal lymphomas. Conclusions of the Workshop held by the EAHP and the SH in Thessaloniki, Greece

**DOI:** 10.1111/j.1365-2559.2006.02369.x

**Published:** 2006-04

**Authors:** E Campo, A Chott, MC Kinney, L Leoncini, CJLM Meijer, CS Papadimitriou, MA Piris, H Stein, SH Swerdlow

**Affiliations:** Department of Pathology, Hospital Clinico de Barcelona Barcelona, Spain; 1 Department of Pathology, Vienna General Hospital, Medical University of Vienna Vienna, Austria; 2 Department of Hematopathology, University of TX Health Science Center at San Antonio San Antonio, TX, USA; 3 Dipartimento de Patologia Umana ed Oncologia, University of Siena Siena, Italy; 4 Department of Pathology, VU University Medical Centre Amsterdam, the Netherlands; 5 Department of Pathology, Aristotelian University of Thessaloniki Thessaloniki, Greece; 6 Programa de Patologia Molecular, Centro Nacional de Investigaciones Oncologicas Madrid, Spain; 7 Department of Pathology, Benjamin Franklin University Hospital Berlin, Germany; 8 Department of Pathology, University of Pittsburgh School of Medicine Pittsburgh, PA, USA

**Keywords:** extranodal lymphomas, cutaneous lymphoma, thyroid lymphoma, ALCL lymphoma

## Abstract

Campo E, Chott A, Kinney M C, Leoncini L, Meijer C J L M, Papadimitriou C S, Piris M A, Stein H & Swerdlow S H (2006) *Histopathology***48,** 481–504

**Update on extranodal lymphomas. Conclusions of the Workshop held by the EAHP and the SH in Thessaloniki, Greece**

Classification and proper treatment of extranodal lymphoma is hindered by the diversity of lymphoma types and the relative rarity of many of these tumour types. In order to review controversial issues in extranodal lymphoma diagnosis, a joint Workshop of the European Haematopathology Association (EAHP) and the Society for Hematopathology (SH) was held, where 99 selected cases were reviewed and discussed. This Workshop summary is focused on the most controversial aspect of cutaneous B-cell lymphoma, other extranodal B-cell lymphomas, plasmablastic lymphoma and anaplastic large-cell lymphoma in extranodal sites, and makes practical recommendations about diagnosis and therapeutic approaches.

## Introduction

Extranodal lymphoma diagnosis, classification and appropriate treatment are a frequent challenge in routine lymphoma diagnosis, due to the significant variety of morphologies, molecular alterations and clinical presentations that the term extranodal lymphoma encompasses. Thus, B- and T-cell lymphoma diagnosed at extranodal sites may imply quite different risks for the patient and require diverse therapeutic approaches.

A workshop was organized by the European Association of Haematopathology (EAHP) and the Society for Hematopathology (SH) during the XII Meeting of the EAHP held in Greece in October 2004, in order to define better the single entities included within the spectrum of extranodal B- and T-cell lymphoma.

Ninety-nine cases were received and reviewed by a panel of experienced haematologists. The Workshop provided a valuable opportunity for an open discussion between case submitters, the panel and other participants, whose main conclusions are summarized in the current report.

## Highlights of cutaneous lymphomas

Several lymphoma cases were presented highlighting the usefulness of the recently published new consensus World Health Organization-European Organization for the Research and Treatment of Cancer (WHO-EORTC) classification of cutaneous lymphoma.^1^ In the group of primary cutaneous B-cell lymphomas (Table 1), the following clinicopathological entities could be differentiated: (i) primary cutaneous marginal zone B-cell lymphoma, (ii) primary cutaneous follicle centre lymphoma (PCFCL), (iii) primary cutaneous diffuse large B-cell lymphoma, leg type (PCLBCL leg-type), and (iv) primary cutaneous large B-cell lymphoma, other, in which primary cutaneous intravascular B-cell lymphoma is included. Since the classification of primary cutaneous large B-cell has been the subject of much debate in the past, they are discussed here in more detail. The basis for these new insights is that the available and new data indicate that PCFCL and PCLBCL, leg-type are indeed different clinicopathological entities with their own immunophenotypical and genotypic characteristics.

**Table 1:** B-cell cutaneous lymphoma. Learning from the Workshop

1	Primary cutaneous follicle centre lymphoma and primary cutaneous diffuse large B-cell lymphoma, leg type, are indeed different clinicopathological entities with their own immunophenotypic and genotypic characteristics
2	Primary cutaneous follicle centre lymphoma is a tumour of neoplastic follicle centre cells, which generally present on the head or trunk. The gth pattern may be follicular, follicular and diffuse or diffuse
3	Lymphomas with a diffuse gth pattern and a monotonous proliferation of centroblasts and immunoblasts are, irrespective of the site of the disease, classified as primary cutaneous diffuse large B-cell lymphoma, leg type, or rarely primary cutaneous large B-cell lymphoma, other
4	The term primary cutaneous large B-cell lymphoma, other, refers to rare cases of large B-cell lymphoma arising in the skin, which do not belong to the group of primary cutaneous diffuse large B-cell lymphomas, leg type, or the group of primary cutaneous follicle centre lymphomas. These cases include morphological variants of diffuse large B-cell lymphoma, such as anaplastic or plasmablastic subtypes or T-cell/histiocyte-rich large B-cell lymphoma

PCFCL is defined as a tumour of neoplastic follicle centre cells, usually a mixture of centrocytes (small and large cleaved follicle centre cells) and variable numbers of centroblasts (large non-cleaved follicle centre cells with prominent nucleoli), which generally present on the head or trunk. The growth pattern may be follicular, follicular and diffuse or diffuse, i.e. a continuum without distinct categories or grades. Lymphomas with a diffuse growth pattern and a monotonous proliferation of centroblasts and immunoblasts are, irrespective of site, excluded and are classified as PCLBCL, leg-type or, rarely, primary cutaneous large B-cell lymphoma, other. However lymphomas with these histological characteristics occur only rarely on head and trunk.

**Figure 1:**
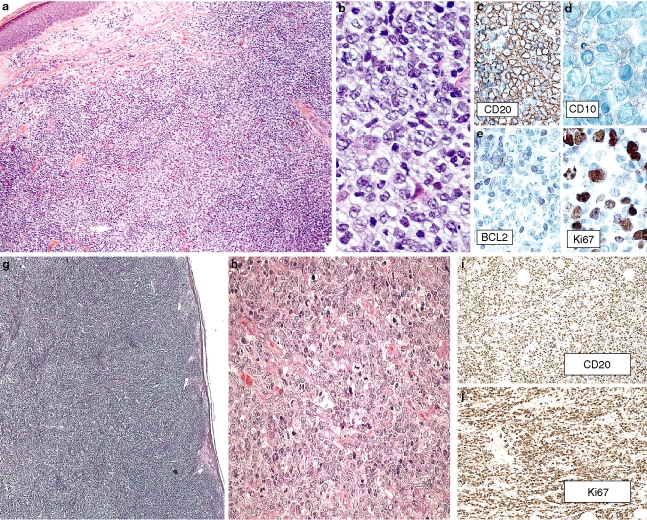
a–f, Primary cutaneous follicle centre lymphoma. **a,** Nodular pattern. **b,** Centroblastic predominance. **c,** CD20. **d,** CD10. **e,** Bcl-2. **f,** Ki67. **g–j,** Primary cutaneous diffuse large B-cell lymphoma, leg type. **g,h,** morphology, H&E. **i,** CD20. **j,** Ki67. Cases contributed by C. Girardet (A6) and R. S. Robertorye (A8).

Irrespective of the growth pattern (follicular or diffuse), the number of blast cells or the presence of either localized or multifocal skin disease, these PCFCLs have an excellent prognosis with a 5-year survival of over 95%. A recent study suggests that strong expression of Bcl-2 in the subset of PCFCL with a diffuse large cell histology and primary cutaneous large B-cell lymphoma, other, is associated with a more unfavourable prognosis.^2^ However, it is important to exclude systemic B-cell lymphoma when Bcl-2 expression is found in PCFCL (Figure 1).

PCLBCL, leg type, shows predominance or confluent sheets of centroblasts and immunoblasts (round cells), characteristically presenting with skin lesions on the (lower) legs. Uncommonly, skin lesions with a similar morphology and phenotype can arise at sites other than the legs.

The 5-year survival of PCLBCL, leg type, is 55% in the Dutch and Austrian cutaneous lymphoma registries. It affects predominantly elderly female patients. PCLBCL, leg type, on the leg has a worse prognosis compared with PCLBCL, leg type, presenting at other sites.^3^ The presence of multiple skin lesions at diagnosis is a significant adverse risk factor. In a recent study, patients presenting with a single skin tumour on one leg had a disease-related 5-year survival of 100%, whereas patients presenting with multiple skin lesions on one or both legs had a disease-related 5-year survival of 45% and 36%, respectively.^4^,^5^

The phenotype of PCFCL is that of a B-cell (CD20+, CD79a+), Bcl-6+, Bcl-2– or faint +, Mum-1/IRF4–, CD10–/+ diffuse lesion mostly negative. The t(14;18) often seen in systemic follicular lymphomas and part of systemic diffuse large B-cell lymphoma (DLBCL) is absent.^6^ In contrast, PCLBCL, leg type, has a B-cell phenotype (CD20+, CD79a+), but is MUM-1/IRF4+ and FOX-P1+. Bcl-6 is expressed by most cases, whereas CD10 staining is generally absent. Bcl-2 is positive, although t(14;18) is absent.^7^–^11^

Chromosomal imbalances have been identified by comparative genomic hybridization (CGH) analysis in a minority of PCFCLs, but a consistent pattern has not yet emerged. In a recent study using interphase fluorescence *in situ* hybridization, no evidence of translocations involving IgH, myc or Bcl-6 loci were found.^12^ PCFCLs have the gene expression profile of germinal centre-like large B-cell lymphomas.^13^

Recent genotypic studies in PCLBCL, leg type, demonstrated translocations involving *myc*, *Bcl-6* and *IgH* genes, but not in patients with a PCFCL with a diffuse infiltration of large centrocytes and suggest that PCLBCL, leg type, has an activated B-cell gene expression profile.^13^–^15^ Inactivation of *p15* and *p16* tumour suppressor genes by promotor hypermethylation has been detected in both PCFCL and PCLBCL, leg type.^8^

PCLBCL, other, refers to rare cases of large B-cell lymphomas arising in the skin, which do not belong to the group of PCLBCL, leg type, or the group of PCFCL.^1^ These cases include morphological variants of DLBCL, such as anaplastic or plasmablastic subtypes or T-cell/histiocyte-rich large B-cell lymphomas. Such cases are generally a skin manifestation of a systemic lymphoma. Rare cases of primary cutaneous T-cell/histiocyte-rich B-cell lymphoma, characterized by the presence of large scattered B cells in a background of numerous reactive T cells, have been reported. Clinically, they show similarities to the groups of PCFCL and primary cutaneous marginal zone lymphoma (PCMZL). These lymphomas commonly present with skin lesions on the head, the trunk or the extremities, and may in fact represent an exaggerated T-cell infiltrate in association with other forms of cutaneous B-cell lymphoma (CBCL). Unlike their nodal counterparts, they appear to have an excellent prognosis. In addition, rare cases of primary cutaneous intravascular large B-cell lymphoma may be included in the category of PCLBCL, other.

In the group of cutaneous T-cell lymphoma, primary cutaneous lymphomas (CTCL) other than mycosis fungoides, Sezary syndrome and primary cutaneous CD30+ lymphoproliferative diseases, which constitute about 10% of all CTCL, have been classified. With few exceptions, these lymphomas are clinically aggressive. Cases with a favourable prognosis are restricted to the provisional entity of small/medium-sized pleomorphic CTCL with a CD4+ T-cell phenotype that presents with localized disease.^16^ One change from prior classifications was prompted by recent studies showing clinical, histological and immunophenotypical differences between cases of subcutaneous panniculitic-like T-cell lymphoma (SPLTCL) with an α/β T-cell phenotype and those with a γ/δ T-cell phenotype, suggesting that these may represent different entities.^17^ Whereas SPLTCL with an α/β T-cell phenotype are homogeneous with a rather indolent clinical behaviour in many patients, SPLTCL with a γ/δ T-cell phenotype overlaps with other types of γ/δ+ T/natural killer (NK)-cell lymphoma and invariably runs a very aggressive clinical course. It was therefore suggested that the term SPLTCL be restricted to SPLTCL with an α/β T-cell phenotype. Three disorders, previously included in the broad group of post-transplant lymphoproliferative disorder (PTLD), unspecified in the WHO classification, are proposed as provisional entities. These include aggressive epidermotropic CD8+ CTCL, cutaneous γ/δ T-cell lymphoma (including cases formerly diagnosed as SPLTCL with a γ/δ phenotype) and primary cutaneous small–medium CD4+ T-cell lymphoma. In the WHO-EORTC classification the term PTL, unspecified, is maintained for remaining cases that do not fit into either of these provisional entities.

## Plasmacytoid dendritic cell tumours

Plasmacytoid dendritic cell tumours are composed of a monotonous proliferation of cells with lymphoblast-like morphology and expression of CD4 and CD56. Because of this immunophenotype it was initially believed that these tumours are derived from NK cells and therefore were categorized in the WHO classification as blastic NK-cell lymphomas. With the availability of new antibodies it could be demonstrated that the cells of this tumour type resemble immunophenotypically cells that were first described under the term ‘lymphoblasts’. These cells were renamed several times according to the features subsequently discovered: T-associated plasma cells, plasmacytoid T cells, plasmacytoid monocytes and finally plasmacytoid dendritic cells. The immunophenotype of these cells is unique in that it is distinct from all known lymphoid and myeloid cell subsets (see Table 2).

**Table 2:** Comparison of the immunophenotype of normal and tumoral plasmacytoid dendritic cells (PDC)

Marker	PDC in reactive lymph nodes	PDC tumours *N* = 7
CD4	+	+
CD56	–/(+)	+
HLA-DR	+	+
CD43	+	+
TCL1	–/(+)	+
CD123	+	+
CD45RA	+	+
CD68	+	+/–
Granzyme B	–	+/–
TdT	–	+/–
Perforin	–	–
CD45RO	–	–
Myeloperoxidase	–	–
CD34	–	–
CD20, CD3	–	–

It was the elucidation of the function of the cells under discussion which clarified their real nature. It was found that these cells have the functional profile of dendritic cells in that they secrete large amounts of interferon (IFN)-α/β, express TLR-7 and TLR-9, and promote the function of NK, B and T cells as well of myeloid dendritic cells.^18^

Therefore, an appropriate name for these cells is ‘plasmacytoid dendritic cells’. Immunologists also call these cells ‘interferon-producing cells’. Since they are not the only cells which produce IFN and since the latter term ignores the other features of these cells, the Workshop board recommended use of the term ‘plasmacytoid dendritic cells’.

Plasmacytoid dendritic cells directly regulate the functional activity of T cells by these functions and thus link innate and adaptive immune responses. Thus, plasmacytoid dendritic cells obviously carry at least parts of the ‘evolutionary immunological memory’, which is important for organisms to be protected by a first-line, immediately effective defence system.

The published immunophenotype of neoplasms of plasmacytoid dendritic cells is nearly identical to that of normal plasmacytoid dendritic cells occurring in lymph nodes (Table 2). Among the neoplasms submitted to the Workshop there were seven cases which had exactly the immunophenotype of plasmacytoid dendritic cell tumours as listed in Table 2. All seven cases also displayed the typical histopathological features of this tumour category, which can be summarized as follows:

- Immature, lymphoblast-like cytology.
- Agranular cytoplasm.
- Cytoplasm full of microvesicles.
- Infiltration of the dermis,
- But consistent sparing of the epidermis.
- Involvement of lymph nodes in the para/interfollicular areas.

The diagnostic separation of plasmacytoid dendritic cell tumours from other haematopoietic malignancies is clinically relevant, since the clinical characteristics and clinical outcome, including response to current treatment modalities, differ from those of lymphomas and myeloid proliferative diseases. Plasmacytoid dendritic cell tumours usually affect elderly patients, manifest in the skin and bone marrow, evolve frequently to an overt leukaemia associated with an aggressive course and death within 3 years despite initial response to therapy. This adverse disease course requires the development of new therapeutic strategies.

## Thyroid lymphoma

Lymphomas of the thyroid are rare. They make up no more than 5% of thyroid neoplasms, no more than 2.5% of lymphomas in general and only up to 7% of extranodal lymphomas.^19^,^20^ About 50–80% of primary thyroid lymphomas are of diffuse large B-cell (DLBCL) type and, in most series, about 20–30% are extranodal marginal zone B-cell lymphomas (MZBCL) of mucosa-associated lymphoid tissue type (MALT lymphoma)^19^,^21^–^23^ (Swerdlow, unpublished). A minority of the DLBCLs also include a MALT lymphoma component. Although in the past many thyroid lymphomas were thought to be of follicular centre cell type,^24^,^25^ with the recognition of MALT lymphomas^25^,^26^ and the segregation of DLBCL, the more recent series have reported no more than 12% follicular lymphomas (FL). Some series of primary thyroid lymphomas have also included rare cases of plasmacytoma (which some would argue represents a MALT lymphoma),^25^,^27^ Burkitt lymphoma, small lymphocytic lymphoma, anaplastic large cell lymphoma and peripheral T-cell lymphoma. At least in some series, the vast majority of thyroid lymphomas are associated with lymphocytic/Hashimoto's thyroiditis. Primary lymphomas of the thyroid show a female predominance and occur in adults with a mean age in the 60s. About 90% of cases are stage IE or IIE, although some report a greater proportion of higher stage cases. The MALT lymphomas are considered indolent neoplasms with disease-specific and even overall 5-year survivals from 60 to 100% whereas patients with DLBCL are reported to have a 40–70% 5-year survival, whether or not they have a MALT component.^19^,^22^,^23^,^28^ Advanced age or stage as well as a diagnosis of DLBCL versus MALT lymphoma are recurrently reported adverse prognostic indicators.^19^,^22^,^23^,^28^

The Workshop included five thyroid lymphomas, none of which was of MALT type. These cases raised a number of both practical and biological issues (Table 3). First, what is a primary thyroid lymphoma? Definitions have included a dominant thyroid lesion, primary complaint related to the thyroid, thyroid involvement at diagnosis or a history of Hashimoto's thyroiditis. Series of secondary lymphomas of the thyroid will include a much wider variety of lymphomas with a smaller proportion of MALT lymphomas and DLBCL.^21^ Nevertheless, one must recognize, as seen in this Workshop, that uncommon lymphomas can be primary in the thyroid. As illustrated in the Workshop, rarely even peripheral T-cell lymphomas can be localized to the thyroid.^29^,^30^ Like many other primary thyroid lymphomas, these also appear to be associated with Hashimoto's thyroiditis. The Workshop case of a mantle cell lymphoma in the thyroid engendered a discussion about the criteria for designating a disseminated lymphoma as being of primary extranodal type, a label of uncertain clinical significance in this setting. Arguments put forward to support a primary designation included a markedly enlarged thyroid, apparent thyroiditis and simultaneous presentation of the extrathyroidal disease. The possibility that lymphomas might be more likely to home to a thyroid that has thyroiditis could also be considered.

**Table 3:** Thyroid lymphoma, pointers from the Workshop

Most common thyroid lymphoma is diffuse large B-cell lymphoma, less often MALT lymphoma, some follicular lymphomas, rarely other types (see below)
Designation of a disseminated thyroid lymphoma as ‘primary’ may be problematic but it is not of known clinical significance
Primary follicular lymphomas occur in the thyroid; however, they are currently probably under-recognized as some cases share many morphological, immunophenotypic and genotypic features with MALT lymphomas. Like the situation with some other types of extranodal FL, this distinguishes a subset of these cases from many nodal FL and suggests that they are a part of a separate and distinct entity; however, it also makes categorization of some individual cases controversial
Lymphoepithelial lesions occur in thyroiditis and varied types of thyroid lymphomas, i.e. they are NOT pathognomonic or even highly suggestive of a MALT lymphoma
Although controversial and in contrast to the situation in the salivary gland, documentation of a major clonal B-cell population in the thyroid strongly favours a B-cell lymphoma over an autoimmune disorder
The newly described *FOXP1/IGH* translocation is important in thyroid MALT lymphomas

MALT, Mucosa-associated lymphoid tissue; FL, follicular lymphoma.

**Figure 2:**
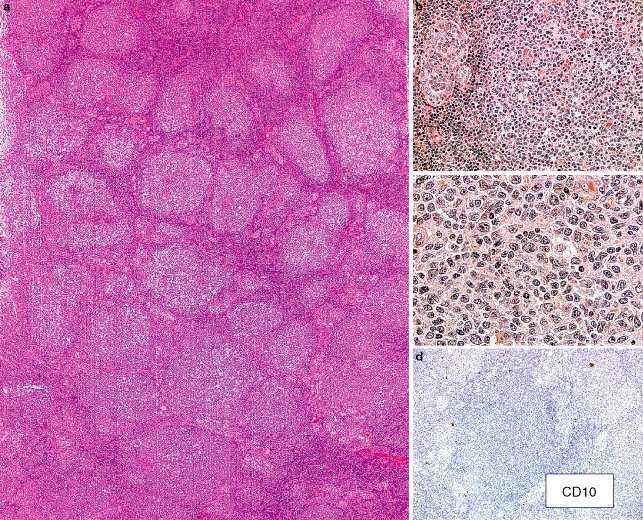
Follicular lymphoma of the thyroid. **a,** The thyroid in this 70-year-old male is extensively infiltrated by numerous lymphoid follicles with relatively homogeneous-appearing follicular/germinal centres. **b,** The interfollicular regions (left) adjacent to the neoplastic-appearing follicle demonstrate many small lymphocytes and prominent lymphoepithelial lesions. **c,** There are numerous small angulated lymphocytes with pale cytoplasm but only infrequent centroblasts in this follicular/germinal centre. (**a–c,** haematoxylin and eosin.) **d,** There are only scattered cells staining distinctly for CD10. Weak positivity was described in the case presentation. (CD10 immunostain with haematoxylin counterstain.) Other immunophenotypic studies were reported to show the following: CD20+, IgM–, IgD–, Bcl-6+, Bcl-2– and 50% Ki67 staining in the follicular structures. Polymerase chain reaction (PCR) studies did not demonstrate a clonal *IGH* rearrangement and neither PCR nor cytogenetic fluorescence *in situ* hybridization studies showed an *IGH/BCL2* translocation. Case contributed by E. Navratil, A. Dogan, P. Isaacson.

The second and probably most contentious issue concerned the criteria for distinguishing FL of the thyroid (with lymphoepithelial lesions) from a MALT lymphoma (with follicular colonization) and led to a broader discussion of whether extranodal FLs are a unique entity. This issue was raised with the presentation of a case that was representative of a study subsequently published in abstract form (Figure 2).^31^ These authors report a series of 22 patients with FL presenting in the thyroid. Many but not all were CD10+ (16/22), all were Bcl-6+ but almost half lacked Bcl-2 expression (9/22) and only half of those studied had an *IGH/BCL2* translocation. *BCL6* translocations were present in 2/12 cases. Morphologically, similarities to MALT lymphomas reportedly included a marked interfollicular expansion and prominent lymphoepithelial lesions. They noted a correlation between the lack of Bcl-2 protein and gene translocation, stage IE disease and a good clinical outcome, suggesting that these are the cases that may be the truly primary thyroid FL. This constellation of findings, together with the morphological features, led some to believe that at least a subset of these lymphomas were, in fact, more like MALT lymphomas. However, while a lack of CD10 may be unusual in other extranodal FL,^32^ Bcl-6 expression is not expected in MALT lymphomas and absent Bcl-2 expression and especially absent *IGH/BCL2* translocations are features more commonly seen in cutaneous and certain other extranodal FL compared with nodal FL.^12^,^32^ This is in contrast to primary FL of the gastrointestinal tract, which is most common in the duodenum and is usually Bcl-2+.^33^,^34^ Also relevant is the observation that CD10 expression is not necessarily pathognomonic of a follicular centre cell lymphoma. There are very rare CD10+ MZBCLs, including a reported thyroid MZBCL^35^ and a Bcl-6– nodal MZBCL.^36^ Although no definite consensus was reached, it should be noted that it is already well documented that classic CD10+, IGH/BCL2+ extranodal FL can closely resemble MALT lymphomas.^37^ At a minimum, while the actual frequency of *bona fide* FL arising in the thyroid remains to be determined, it clearly does occur. Furthermore, it was briefly discussed that the thyroid DLBCL presented at the workshop was CD10+, as were almost half of the DLBCLs of the thyroid we had reviewed, with 1/2 tested even demonstrating an *IGH/BCL2* translocation. This suggests that a subset of DLBCLs of the thyroid is also of germinal centre cell type, inferring that it would not be unexpected to find FL at the same site.

**Figure 3:**
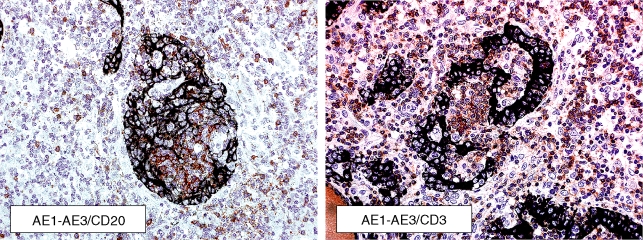
Classical Hodgkin lymphoma in thyroid. These double immunostains with the AE1/AE3 cytokeratin stain in black demonstrate that the lymphoepithelial lesions present in this case included some with many B cells (CD20 immunostain in red/brown on the left) and some with many T cells (CD3 immunostain in red/brown on the right). (Double immunostains with haematoxylin counterstain.) Case contributed by M. Vornanen.

Third, the Workshop cases highlighted the observation that lymphoepithelial lesions (LELs) are not pathognomonic of a MALT lymphoma. Aside from LELs being a part of Hashimoto's thyroiditis, morphologically identified LELs were present in varied B-cell and even non-B-cell lymphomas. LEL demonstrating thyroid follicles stuffed with lymphoid cells (‘MALT ball’ LEL^19^) as well as large LELs are reported to be characteristic of those found in MALT lymphomas;^19^,^20^,^38^ however, morphologically similar-appearing LELs are described in some T-cell neoplasms.^30^ MALT lymphomas also demonstrate LELs that are more like those seen in the salivary gland without intra-acinar balls of neoplastic lymphoid cells. While finding a paucity of B-cells will often help exclude the type of LEL seen in MALT lymphomas, the case of Hodgkin's lymphoma had LEL that included areas with numerous B cells (Figure 3). In addition, although many of the LELs in Hashimoto's thyroiditis are associated predominantly with T cells, rare small B-cell LELs were reported to occur in 13/40 cases in one series and another smaller series describes predominantly B cells in the LELs of even ‘early’ Hashimoto's thyroiditis.^20^,^38^,^39^ It should also be remembered that LELs in a DLBCL do not make it of ‘MALT type’.

Given that Hashimoto's thyroiditis, which can demonstrate a very dense lymphoplasmacytic infiltrate, and LELs can be very difficult to distinguish from MALT (or other) lymphomas, the question was raised as to whether, unlike in the salivary gland,^40^–^42^ genotypic studies can be used to distinguish thyroiditis with LEL from a MALT lymphoma. While the literature is not consistent, Hsi *et al*. and others have reported no evidence of B-cell clones in Hashimoto's thyroiditis.^38^,^43^ Of interest, in a recent study reporting ‘clonal B cell populations in a minority of patients with Hashimoto's thyroiditis', the polymerase chain reaction (PCR)-identified clones were not only associated with a polyclonal smear but were not reproducible when PCR was performed on either deeper sections in the same block or using another block.^44^

Finally, although not addressed in this session, the subject of chromosomal abnormalities in MALT lymphomas was covered.^45^ It is of interest that the newest chromosomal translocation to be associated with MALT lymphomas, t(3;14)(p14.1;q32) involving *IGH* and *FOXP1*, has been reported in 10% of MALT lymphomas in general, but has been found in 3/6 cases in the thyroid.^46^

## Lymphomas at other extranodal sites

A total of 21 cases of mostly B-cell lymphomas restricted to unusual sites of involvement (bone, brain, lung, salivary glands, tonsil, testis, prostate, breast, ovary and uterus) were included. The main criteria and reasons for submitting a case were histological classification, uncommon location, differential diagnosis between lymphomatous and non-lymphomatous lesions, as well as discrimination of subgroups and entities with histological, clinical and prognostic importance.

**Figure 4:**
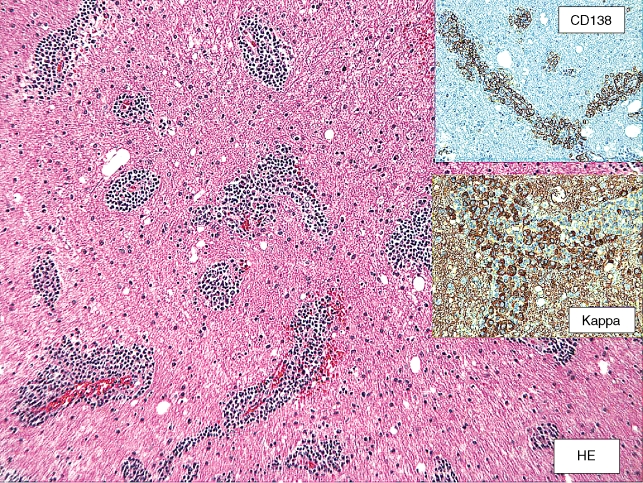
Lymphoplasmacytic lymphoma, primary in brain. Case contributed by E. Hsi (case B5).

Primary small lymphocytic lymphomas of the CNS are very rare. Especially, primary lymphoplasmacytic lymphomas of the brain are extemely uncommon (Figure 4). In such cases secondary infiltrations by systemic lymphoplasmacytic lymphoma, the so-called Bing and Neel syndrome, as well as the possibility of meningeal MALT-type lymphoma of the CNS must be considered seriously.^47^,^48^

Osteolytic and tumorous bone lesions occur secondarily in hairy cell leukaemia.^49^,^50^ Their manifestation before the development of the classical haematological picture of hairy cell leukaemia is extraordinarily rare. Immunohistochemical positivity of neoplastic cells for cyclin D1 and annexin 1^50^ could be helpful for the differential diagnosis of such cases of hairy cell leukaemia from other types of bone marrow lymphomas.

Burkitt lymphomas restricted to one organ such as the liver, prostate, testis and uterus are very rare.^50^–^53^ Their accurate diagnosis based on morphological, immunohistochemical and molecular criteria is necessary because they may have a rather favourable outcome after appropriate therapy.^51^,^53^

**Figure 5:**
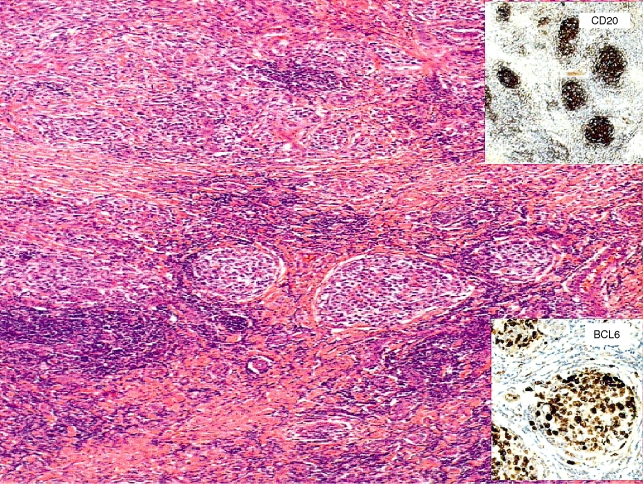
Testicular follicular lymphoma, grade IIIa, t(14;18)–. Case contributed by C. Bacon and A. Dogan (case G6).

Primary extranodal, extracutaneous FLs occur rather infrequently.^54^–^58^ In general, with the possible exception of gastrointestinal FL, these lymphomas have microscopic, immunohistochemical and molecular features different from the nodal FL, being in most cases Bcl-2– and t(14;18)–.^56^,^59^–^62^ One of the cases discussed in the Workshop illustrated a recurrence in the salivary gland of a case initially diagnosed as cutaneous follicular centre lymphoma, which could point to the existence of some common features for most of these extranodal FLs. Nevertheless, some extranodal localizations need to be considered as separate subgroups, as happens, for example, with the testicular FLs of childhood, which seem to have an excellent outcome even following simple surgical resection^55^,^61^). A few similar cases may also occur in adults (Figure 5).

## Large B-cell lymphomas of terminally differentiated B cells

Plasmablastic lymphoma is considered a variant of DLBCL in the WHO classification. This tumour was initially described as a DLBCL with immunoblastic or large B cells and a phenotype resembling the terminally differentiated B cell, characterized by negative or weak expression of mature B-cell markers (CD20) and positivity for plasma cell-associated antigens (CD38, CD138).^63^ The tumours were mainly diagnosed in patients infected with the human immunodeficiency virus (HIV) and they presented commonly in the mucosa of the oral cavity. The clinical behaviour was aggressive with poor response to therapy and short survival. In spite of this initial definition, the term ‘plasmablastic’ has been applied in different situations to describe morphological or immunophenotypic features in the context of other large B-cell or plasma cell neoplasms. In addition, several studies have now recognized other lymphomas with similar morphological and phenotypic plasmablastic features but with diverse clinical and molecular characteristics suggesting that they may correspond to different disease entities (Table 3). Several cases submitted to the Workshop illustrated the diversity of these tumours and provide new insights into their clinical and pathological characteristics.

### Plasmablastic lymphoma

**Figure 6:**
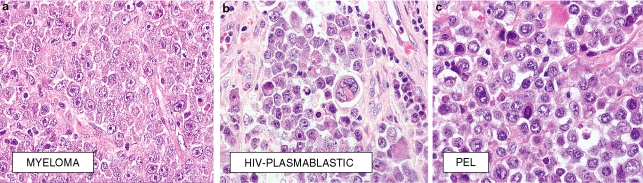
**a,** Extramedulary involvement by multiple myeloma. Case A11 contributed by F. Fend. **b,** Plasmablastic lymphoma with plasmacytic differentiation in a HIV+ patient. Case C12 contributed by J. Teruya-Feldstein. **c,** Primary effusion lymphoma presenting as a solid tumour. The tumour was positive for HHV8. Case C7 contributed by A. Dogan.

Plasmablastic lymphomas were initially recognized as proliferations of large B cells with immunoblastic morphology and plasma cell immunophenotype. The cells had a cohesive pattern of growth and a monomorphic appearance with little variability in terms of size and cytoplasmic characteristics. The tumours presented in the oral cavity and were mainly diagnoses in HIV+ patients.^63^ Three cases presented in the Workshop were diagnosed as plasmablastic lymphoma in HIV+ patients. However, these three cases had morphological and clinical differences from the original description indicating that the spectrum of these lymphomas may be broader than initially considered. The three tumours were morphologically characterized by a proliferation of immunoblasts and plasmablasts that were CD20– and CD79a– but positive for plasma cell-associated antigens and Epstein–Barr virus (EBV) with a type I latency pattern. However, in contrast to the original description of the plasmablastic lymphoma of the oral cavity, they contained also a range of cells with plasmacytic differentiation similar to the subtype recently recognized by Colomo *et al*. as plasmablastic lymphoma with plasmacytic differentiation^64^(Figure 6). These tumours presented in extranodal sites other than oral mucosa (rectal mucosa 2, testis 1). One of the cases had a low M-spike and bone marrow involvement. This case illustrates the recent findings by Teruya-Feldstein *et al*. indicating that plasmablastic lymphoma may present with extensive bone involvement and a minimal M-component.^65^ However, the clinical and pathological characteristics were not consistent with multiple myeloma (MM). The behaviour of the plasmablastic lymphoma is usually aggressive. However, recent reports suggest that patients treated now with highly active antiretroviral therapy (HAART) and improvement of their CD4 counts may have a better outcome than the cases initially reported.^65^,^66^

### Primary effusion lymphomas and extra-cavitary primary effusion lymphoma variant

Primary effusion lymphoma (PEL) is a well-recognized entity in immunosuppressed patients associated with HHV-8 and, frequently, EBV infection. It is characterized by a proliferation of large pleomorphic B cells that lack expression of B-cell markers and immunoglobulin but commonly express plasma cell-associated antigens and CD30. These tumours usually present as effusion lymphomas with no involvement of tissues.^67^ However, some patients may develop a solid tissue mass in the evolution of the disease or, alternatively, may present with a tissue tumour mass and subsequently develop a cavitary lymphomatous effusion. The extracavitary dissemination usually includes lymph nodes, lung, skin, gastrointestinal tract and occasionally oral mucosa and larynx.^68^,^69^ One of the cases presented in the Workshop illustrated this presentation as a HHV-8+ nodal large cell lymphoma with plasmablastic features that subsequently developed a gastrointestinal mass and tumoral ascites. Several studies have recognized also a pure extracavitary variant of PEL in which the tumour presents as a solid tissue mass without evidence of malignant effusions.^68^,^70^ This variant has similar morphological, immunophenotypic and molecular characteristics to PEL, suggesting that they belong to the same entity. The extracavitary presentation of these tumours also involves similar sites such as skin, gastrointestinal tract and lymph nodes. However, these solid tumours have some phenotypic peculiarities including a slightly more frequent expression of mature B-cell markers (CD20) and immunoglobulins, occasional expression of T-cell-associated markers and less EBV positivity. Interestingly, patients with solid variants of PEL may have fewer opportunistic infections and better survival than those with classical PEL. However, similar to the recent observation in plasmablastic lymphoma,^65^ this better outcome may be due in part to the treatment of HIV+ patients with HAART leading to better control of the HIV infection and improvement of the CD4 counts.^68^

### Extramedullary plasmablastic tumours secondary to plasma cell neoplasms

Plasmablastic lymphomas, particularly with plasmacytic differentiation, are morphologically and phenotypically similar to extramedullary large cell transformation of plasma cell neoplasms. The distinction between these neoplasms may be difficult if not impossible without clinical correlation. Most transformations of extramedullary plasmacytomas or extramedullary dissemination of MM occur as secondary evolution of these diseases several years after diagnosis but in some cases extramedullary involvement may occur simultaneously with the initial diagnosis of MM.^65^,^71^ One of the cases submitted to the Workshop was an extranodal large B-cell neoplasm with plasmablastic features which 2 years later, and after a complete remission with chemotherapy, progressed to an overt MM, illustrating the difficulties in the differential diagnosis between plasmablastic lymphoma and high-grade plasma cell neoplasms. Clinically, extramedullary plasmablastic tumours secondary to plasma cell neoplasms occur in immunocompetent patients, whereas plasmablastic lymphomas are usually associated with immunosuppression. Phenotypically, both types of tumours are relatively similar, although CD56 expression may be slightly more common in the first group and cyclin D_1_ positivity is detected only in MM patients.^65^,^71^,^72^ EBV infection is frequently present in plasmablastic lymphoma and usually negative in plasma cell tumours. However, occasional nasopharyngeal plasmacytomas and derived plasmablastic lymphomas may be EBV+.^65^

### Other large b-cell lymphomas with plasmablastic features

In addition to the previous types of tumours, other lymphomas may have some features like those of plasmablastic lymphoma yet they may represent different entities. Particularly, DLBCL expressing anaplastic lymphoma kinase (ALK) protein may correspond to a distinct disease in this spectrum of neoplasms.^73^ These tumours are usually nodal and present in young immunocompetent patients. Morphologically, they are composed of monomorphic proliferations of large B cells, EBV–, with a plasmablastic phenotype and frequent expression and secretion of IgA. Similar to anaplastic large-cell lymphoma, ALK expression is due to ALK rearrangements, particularly with clathrin or less frequently with nucleophosmin.^74^,^75^ The behaviour is aggressive and most patients die of the disease in a short period of time. Another tumour in this spectrum is the HHV-8+ large B-cell lymphoma emerging in the context of multicentric Castleman's disease.^76^ This tumour has been named plasmablastic lymphoma because of the immunoblastic/plasmablastic appearance of the cells. However, the cells strongly express CD20, a marker uncommon in the late stage of B-cell differentiation. Occasional aggressive DLBCL shows marked secretory differentiation with abundant basophilic cytoplasm, a paranuclear hof and marked cytoplasmic immunoglobulin expression. However, these tumours are strongly CD20+, EBV– and occur in immunocompetent patients.^77^ These lymphomas, as well as the EBV+ DLBCL associated with pyothorax, are probably variants of DLBCL at a more mature terminal stage of differentiation than plasmablastic lymphoma (Table 4).^78^

**Table 4:** Diffuse large B-cell lymphomas (DLBCL) with terminally differentiated B-cell phenotype

Plasmablastic lymphomas (PBL)
Plasmablastic lymphoma of oral mucosa type
Plasmablastic lymphoma with plasmacytic differentiation
HHV-8-associated lymphomas
Plasmablastic lymphoma associated with multicentric Castleman's disease*
Primary effusion lymphoma (PEL)
Extracavitary variant of PEL
Extramedullary plasmablastic tumours secondary to plasma cell neoplasms
Diffuse large B-cell lymphoma expressing ALK
Differential diagnosis*
Diffuse large B-cell lymphoma with secretory differentiation
Pyothorax-associated lymphoma

* These tumours express strong CD20 and therefore are probably better designated as immunoblastic variants of diffuse large B-cell lymphoma.

In summary, the cases presented in the Workshop in the plasmablastic lymphoma session illustrate the clinical and pathological heterogeneity of these lymphomas. These observations are concordant with several recent publications suggesting that large B-cell lymphomas with features of terminally differentiated B cells are different from conventional DLBCL and most probably include different disease entities that should be individually recognized.

## Other lymphoproliferative disorders in immunocompromised patients and EBV-related lymphomas

The WHO classification of tumours of haematopoietic and lymphoid tissues includes immunodeficiency-associated lymphoproliferative disorders (IDA-LPDs)^79^ which are grouped in four main categories: (i) associated with primary immune disorders (PID), (ii) associated with HIV infection, (iii) post-transplant and (iv) methotrexate-associated.

Thirteen examples of extranodal lymphoid proliferation in immunosuppressed patients were submitted to the Workshop, including cases of PTLD; patients with autoimmune disorders treated with methotrexate; patients with a previous lymphoma treated with chemotherapy, and associated with EBV infection without clinical evidence of immunosuppression. HIV+ cases have already been discussed in the plasmablastic lymphoma section.

These cases illustrate the variety of clinical situations in which IDA-LPDs may occur, which includes not only the well-known conditions, but autoimmune disorders, methotrexate or other immunosuppressive treatment, haematological neoplasms and newly emerging phenomena, such as senile LPD,^80^ which may not yet be recognized. In fact, LPDs may be related to one or more underlying viral infections, such as those sustained by EBV, in which there is no recognized cause for immunosuppression.

The IDA-LPDs presented at the Workshop showed heterogeneous pathological manifestations. They included overt malignant lymphomas (of peripheral B- and T-cell derivation), polymorphic (polyclonal and monoclonal) proliferations and lesions of uncertain biological significance that are not different from those observed in nodal disorders.^81^ In particular, among the PTLDs three cases showed plasmablastic/plasmacytic differentiation similar to the previously discussed cases of large B-cell lymphoma with plasmablastic features. About half of the PTLD cases were EBV– and tended to occur later than EBV+ ones. (Interestingly, one case associated with methotrexate was a lymphoproliferative disorder with the morphological features of a Hodgkin-like lesion according to the WHO classification, but the immunophenotype and very aggressive clinical course indicated that it was a true malignant Hodgkin's lymphoma.)

## Mucosa-associated lymphomas

Two major types of non-Hodgkin's lymphomas primarily present at mucosal sites: one is extranodal MZBCL of mucosa-associated lymphoid tissue (MALT lymphoma) which commonly arises in the stomach from mucosal lymphoid tissue that is acquired usually as a reaction to *Helicobacter pylori* and follows an indolent clinical course; the other is represented by enteropathy-type T-cell lymphoma (ETCL) derived from intestinal intraepithelial T cells often evolving as a consequence of adult-onset coeliac disease with usually rapidly fatal outcome. A third, albeit reactive process of the MALT, termed atypical marginal zone hyperplasia of MALT, was described most recently to occur in the tonsil or appendix in childhood. All of these three conditions were represented at the Workshop by at least one case each and merit attention as outlined below. The main focus was on extragastric MALT lymphoma, as gastric cases have been excluded.

### Extragastric malt lymphoma

**Figure 7:**
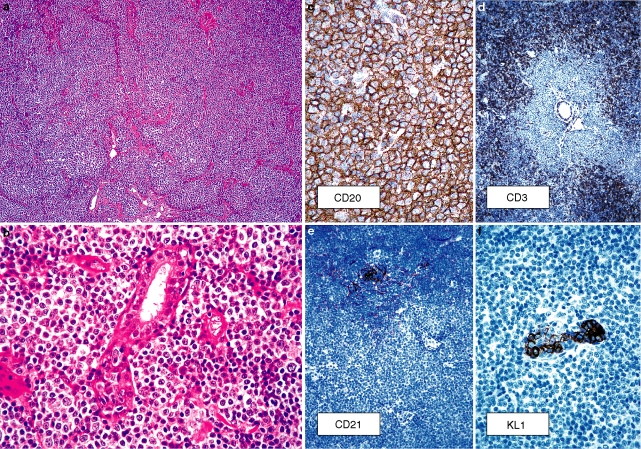
Marginal zone B-cell lymphoma in the breast. **a,b,** Morphology. **c,** CD20. **d,** CD3. **e,** CD21. **f,** KL1. Case contributed by F. Gaillard (D12).

Primary extragastric MALT lymphoma preferentially involves the ocular adnexa, salivary gland, skin, lung, intestine, thyroid and breast^82^–^84^(Figure 7). Recent evidence suggests that extragastric MALT lymphoma may also be associated with infectious agents such as *Borrelia burgdorferi* in cutaneous lesions, *Chlamydia psittaci* in MALT lymphoma of the ocular adnexa, and *Campylobacter jejuni* in a special form of intestinal MALT lymphoma termed immunoproliferative small intestinal disease (IPSID).^85^–^87^ The pathogenic role of these microbes in the development of the respective MALT lymphoma appears, however, to be not as straightforward as that of *H. pylori.*^88^ In general our knowledge of extragastric MALT lymphomas lags somewhat behind that of gastric disease. Therefore this summary concentrates on some general and some new issues in extragastric MALT lymphomas.

Besides the most common sites, Workshop MALT lymphoma cases also comprised unusual examples such as tumours arising in the thymus, subcutaneous tissue, dura and larynx, indicating that there is almost no organ or tissue which, upon triggering by an inflammatory response, cannot give rise to this type of marginal zone B-cell-related neoplasia. Hence, the histological appearance of MALT lymphoma is markedly influenced by the local environment (mucosal versus non-mucosal) and the underlying inflammatory/autoimmune processes. Whereas the cytomorphology of the tumour cells may vary among MALT lymphomas and even within the same tumour, ranging from small lymphoid cells to centrocyte-like cells and monocytoid cells, non-neoplastic germinal centres or colonized remnants thereof are an almost constant, site-independent finding.^82^ At mucosal sites the frequency of LELs, reflecting the interaction between lymphoma cells and epithelial structures, depends heavily on the site involved, i.e. they are invariably present in MALT lymphomas of the thyroid, often seen in salivary glands and lung, rarely encountered in the ocular adnexa, particularly the lacrimal gland, intestine and breast, and exceedingly rare to absent in cutaneous MALT lymphomas. Importantly, LEL are neither specific for MALT lymphoma nor required for the diagnosis.

As MALT lymphoma derives from postgerminal centre memory B cells, it is not surprising that plasmacytic differentiation may occur in up to 30% of extragastric MALT lymphomas and is very often associated with serum paraproteinaemia.^89^ Histologically, small groups or sheets of light chain restricted lymphoplasmacytoid cells and/or plasma cells, usually containing IgM, are part of the infiltrate, occasionally showing intranuclear periodic acid–Schiff-positive material (Dutcher bodies).

Differential diagnostic considerations include small B-cell lymphocytic proliferations, mantle cell lymphoma and FL. Most cases are CD20+, CD5–, Bcl-6–, CD10–, CD23–, and these cells often colonize reactive germinal centres which display a disrupted network of CD21+ follicular dendritic cells. Unfortunately, there is no highly characteristic marker for MALT lymphoma, such as cyclin D_1_ for mantle cell lymphoma. A few cases have been reported to express CD5, some are known to be CD43+, although the frequency of the latter is probably lower than in gastric MALT lymphomas.^90^ Nuclear Bcl-10 expression has been demonstrated to be strong in t(1;14)(q22;q32)+ cases and moderate in almost all t(11;18)(q21;q21)+ MALT lymphomas.^83^ In daily practice, however, the immunohistochemical detection of Bcl-10 expression cannot be recommended as a reliable tool for the demonstration of the t(11;18)(q21;q21).

The remarkable recent advances in genetics and molecular biology in MALT lymphomas^83^ were clearly visible in the Workshop, as half of the submitted MALT lymphoma cases had been investigated for genetic aberrations. Three structural genetic aberrations, t(11;18)(q21;q21), t(1;14)(q22;q32) and t(14;18)(q32;q21), are regarded as specific for MALT lymphoma and their prevalence has recently been evaluated in a large series comprising both gastric and extragastric cases.^45^ More recently, a fourth translocation, t(3;14)(p14.1;q32), has been described as occurring in MALT lymphomas.^46^ Common to all these four aberrations is that their occurrence is markedly influenced by lymphoma site.^45^ The t(11;18)(q21;q21) is preferentially detected in gastric (24%) and pulmonary tumours (50%) but not in ocular adnexal/orbital MALT lymphomas in which the t(14;18)(q32;q21) is found in 24% of cases. The t(3;14)(p14.1;q32) has been detected in three of six thyroidal MALT lymphomas and in 20% of ocular adnexal/orbital tumours.^45^ The very rare t(1;14)(q22;q32) is largely restricted to pulmonary and gastric lesions. Numerical aberrations, such as +3, +12 and +18, may occur isolated or, except in case of a t(11;18)(q21;q21), in combination with a structural aberration. All these genetic aberrations can be detected in routinely processed tissue by fluorescence *in situ* hybridization (FISH); however, the t(11;18)(q21;q21) is usually evaluated by reverse transcriptase (RT)-PCR.^91^ Importantly, both techniques work well with routinely processed tissue.

Are MALT lymphoma-associated genetic aberrations diagnostically helpful? The strong association of the translocations with MALT lymphoma could be helpful in certain diagnostic situations, for instance the demonstration of the t(11;18) by RT-PCR in case of a small lung biopsy with a lymphoid infiltrate suspicious but not diagnostic of MALT lymphoma. Similarly, the close association of trisomies 3 and 18 with MALT lymphoma may be helpful in doubtful cases. As shown in Table 5, the two trisomies, particularly trisomy 3, very often occur in intestinal, salivary gland and ocular adnexal tumours. In context with the appropriate histopathological and immunophenotypic features, the detection of one of these genetic aberrations would strongly favour the diagnosis of MALT lymphoma. Therefore, in the above-mentioned context, the two techniques, i.e. FISH and RT-PCR, are more powerful than immunoglobulin gene rearrangement studies by PCR because they are not only able to detect a monoclonal process but additionally help to classify the disease.

**Table 5:** Frequencies (%) of trisomies 3 and 8 in mucosa-associated lymphoid tissue lymphomas of different sites^45^

	*n*	Trisomy 3	Trisomy 18
Stomach	71	11	6
Skin	51	20	4
Salivary gland	42	55	19
Ocular adnexa/orbit	37	38	14
Intestine	16	75	25
Lung	15	20	7

So far there is no evidence that any of the above-mentioned genetic aberrations is of clinical significance in extragastric MALT lymphomas; however, final conclusions have to await the results of larger series.

Clinically, extragastric MALT lymphomas often involve multiple sites at presentation or recur at distant MALT sites during the course of the disease.^92^ Therefore, thorough staging after diagnosis is very important.

### Enteropathy-type t-cell lymphoma

The diagnosis of ETCL may be challenging, particularly in the case of small endoscopic biopsies and if the lymphoma component is masked by a heavy inflammatory infiltrate. Refractory coeliac disease frequently precedes the diagnosis of ETCL.^93^ In this scenario duodenal biopsies show villous atrophy and a marked increase of cytologically normal, but immunophenotypically abnormal intraepithelial lymphocytes (IELs), usually characterized by loss of CD8. Furthermore, the IELs are monoclonal by PCR. Multiple flat intestinal ulcers may develop, known as ulcerative jejunitis, already containing a few cytologically transformed cells which may be very hard to detect microscopically. The accumulation of immunophenotypically abnormal, monoclonal IELs appears to be the first step in the genesis of ETCL. Patients with refractory coeliac disease and/or ulcerative jejunitis are therefore suffering from a neoplastic T-cell disorder, probably involving most of the gastrointestinal tract.^93^

### Atypical marginal zone hyperplasia of malt

MALT lymphomas usually arise at sites of acquired MALT and are uncommon in native MALT such as tonsil and Peyer's patches. Malignancy in these indolent lymphomas is often inferred by immunoglobulin light-chain restriction and in some cases by ‘aberrant’ expression of CD43. A notable exception to this rule has recently been reported by Attygalle *et al*.^94^ and was discussed at the Workshop. The authors describe six children with marginal zone hyperplasia affecting the tonsil or appendix, characterized by expansion of the marginal zone by centrocyte-like and transformed cells heavily spilling into the tonsillar crypt epithelium mimicking MALT lymphoma. Immunophenotypically, these cells showed λ light-chain restriction and CD43 expression; however, comprehensive molecular studies convincingly failed to show any evidence of monoclonal immunoglobulin gene rearrangement in any of the six cases. Clinical follow-up did not show any recurrence at a mean follow-up of almost 3 years.

## Anaplastic large-cell lymphoma and its differential diagnosis

Anaplastic large-cell lymphoma is a pleomorphic large cell lymphoma with strong expression of the activation antigen CD30 (Ki1) in virtually every cell and frequent involvement of lymph node sinuses.^95^ Anaplastic large-cell lymphoma occurs predominantly in children and young adults, but a second peak incidence is seen in 60–80-year-olds.^96^ In 40–80% of anaplastic large-cell lymphomas, dysregulation of the receptor tyrosine kinase gene *ALK*^97^ on chromosome 2p23 is important in its pathogenesis. ALK expression correlates with young age, systemic disease, a cytotoxic epithelial membrane antigen (EMA)+ phenotype and usually a good prognosis.^98^

Although anaplastic large-cell lymphoma represents only 2–3% of lymphomas overall, a significant number of cases (approximately 40–65%) have extranodal disease either at a primary site or as part of a systemic process.^96^,^99^,^100^ Affected sites include skin (15–30%), bone (5–20%), liver (5–10%), lung (5–15%), soft tissue (10–20%), muscle (< 5%) and rarely (< 1%) gut, testis, parotid, thyroid, breast, pancreas, oral cavity and ocular adnexa. As the differential diagnosis of CD30+ infiltrates includes reactive processes and large cell neoplasms it is crucial to diagnose anaplastic large-cell lymphoma accurately, particularly at extranodal sites where other tumours may be more common. The determination of the extent of extranodal anaplastic large-cell lymphoma (primary versus systemic process) is also critically important, particularly in the skin. In the EAHP Lymphoma Workshop, anaplastic large-cell lymphoma was the diagnosis or part of the differential in eight of the 100 cases submitted. These cases highlighted potential diagnostic pitfalls related to ectopic antigen expression, showed ALK expression does not always correlate with systemic disease and good prognosis and prompted discussion of whether ALK– anaplastic large-cell lymphoma is distinct from peripheral T-cell lymphoma unspecified.

### Morphological or immunophenotypic features that could lead to the wrong diagnosis in anaplastic large-cell lymphoma

**Figure 8:**
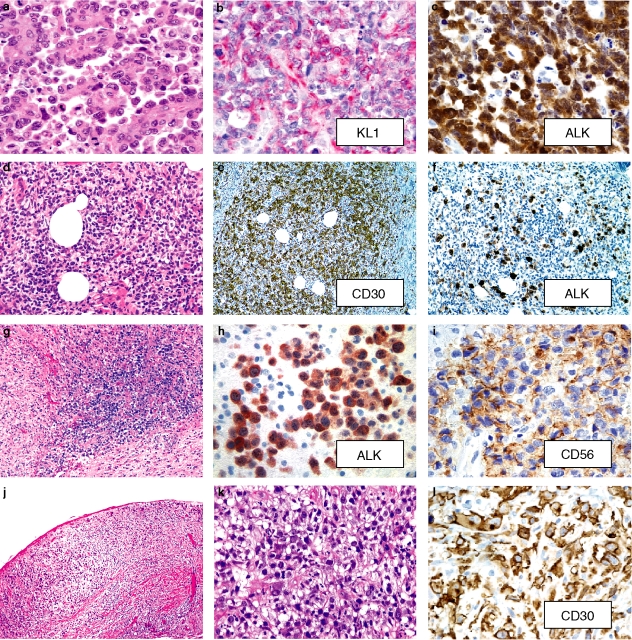
**a–c,** Keratin+ ALK+ anaplastic large-cell lymphoma in a pelvic mass from a 37-year-old female. **a,** The growth pattern suggests an epithelial process.**b,** The large cells strongly express pan keratin (KL1); epithelial membrane antigen is positive and CD45 and T-cell antigens and T-cell receptor gene rearrangements are negative (not shown). Ultrastructural features are compatible with lymphoma. **c,** ALK present in the nucleus and cytoplasm. Case contributed by L. Donner, Temple, TX, USA (B4).^13^ **d–f,** ALK+ primary cutaneous anaplastic large-cell lymphoma presenting as a 30-mm tender thigh nodule in a 26-year-old male (Images case 89,A1). **d,** Sheets of large pleomorphic lymphocytes are present throughout the dermis. Many cells have a hallmark appearance with folded indented nuclei.**e,** The cells strongly express membrane and cytoplasmic (Golgi, dot-like) CD30. **f,** The cells have strong nuclear and cytoplasmic ALK expression. Fluorescence *in situ* hybridization studies confirmed translocation of the *ALK* gene. Careful staging and follow-up at 9 months showed no other evidence of disease. (Case contributed by B. Kim, Pittsburgh, PA, USA).**g–i,** ALK+ primary CNS anaplastic large-cell lymphoma in a 27-year-old HIV– male (Images case 90,B9). **g,** Sheets of dysplastic large lymphocytes diffusely infiltrate the brain. **h,** The tumour cells have cytoplasmic and some nuclear ALK expression and are CD30+ (not shown). **i,** CD56 is present in the tumour cells. (Case contributed by Robert F. Bradley and Michael W. Beaty, Winston Salem, NC, USA.)**j–k,** ALK– anaplastic large-cell lymphoma arising in the fibrous capsule surrounding a breast implant in a 49-year-old female (Images case 91,D11). **k,** Sheets of large tumour cells are present. **l,** The tumour cells are strongly CD30+ with a membrane and Golgi pattern of immunoreactivity. (Case contributed by Daphne de Jong, Amsterdam, the Netherlands).

The differential diagnosis of anaplastic large-cell lymphoma includes other CD30+ large cell proliferations such as viral infection, drug reactions, other lymphomas (Hodgkin's lymphoma and B-, T- and NK-cell lymphomas), carcinoma, melanoma and granulocytic sarcoma.^101^–^104^ In these other processes, CD30 is often present in only a subset of cells and the staining pattern may be weak with a focal or diffuse cytoplasmic pattern rather than the strong membrane and Golgi CD30 expression seen in virtually every cell in anaplastic large-cell lymphoma. In anaplastic large-cell lymphoma, the cohesive growth pattern, lack of leucocyte common antigen (CD45RB) in up to 38%,^105^ frequent EMA expression, null phenotype (10–20% of cases), rare keratin expression (particularly broad-spectrum antikeratin antibody KL1)^106^ make carcinoma a strong consideration, as illustrated by a Workshop case of a CD30+ large pelvic mass in a 37-year-old female with all these features. ALK expression and lack of ultrastructural features of an epithelial malignancy confirmed anaplastic large-cell lymphoma (Figure 8).^107^ In ALK– anaplastic large-cell lymphoma with a null phenotype, complete immunophenotyping, clinical staging and ultrastructural studies may be required to make a definitive diagnosis.^108^ In addition, it should be remembered that ALK expression alone is not diagnostic of anaplastic large-cell lymphoma, as ALK can be present in other tumours, principally inflammatory myofibroblastic and other soft tissue tumours, tumours of neural origin (neuroblastoma, glioblastoma) and a very rare ALK+ B-cell lymphoma.^73^,^97^,^109^,^110^

Expression of myeloid antigens (CD13 and rarely CD117) as detected by flow cytometry is an uncommon feature of anaplastic large-cell lymphoma, and can also be potentially misleading.^111^–^114^ Such a case of a 35-year-old female with a parotid mass was included in the Workshop. Fine-needle aspiration with flow cytometry revealed expression of HLA-DR and CD13 but a lack of CD2, CD3, CD4, CD8, suggesting the diagnosis of chloroma. Excisional biopsy and paraffin immunoperoxidase studies revealed the cells were CD30+, EMA+, ALK-1+, and the correct diagnosis of anaplastic large-cell lymphoma was made. In CD13+ cases, care must be taken to rule out a myeloid process as CD30 can be expressed in granulocytic sarcoma.^103^ Rare cases of myeloid antigen-positive tumours with features of anaplastic large-cell lymphoma (CD30+, EMA+, ALK+), CD56 expression, and a *TPM4 (tropomyosin 4)-ALK* fusion have been described.^115^

### ALK Expression in anaplastic large-cell lymphoma as a determinant of systemic disease and prognosis

As a general rule, ALK+ anaplastic large-cell lymphoma is associated with systemic disease and a better prognosis, but there are important exceptions.^98^,^99^,^116^–^118^ Skin is the most common location of extranodal anaplastic large-cell lymphoma and a critical site to determine with certainty if disease is limited to the skin or secondary to a systemic process. Primary cutaneous anaplastic large-cell lymphoma is indolent (disease-related 5-year survival of > 90%) compared with skin involvement in systemic disease (5-year survival of < 45%).^16^,^119^,^120^ The treatment of primary cutaneous anaplastic large-cell lymphoma is conservative with resection with or without irradiation; low-dose methotrexate is used in multifocal disease not amenable to localized therapy. In contrast, systemic anaplastic large-cell lymphoma requires multiagent chemotherapy. At the present time, there are no reliable biological markers to distinguish the two types of anaplastic large-cell lymphoma. Most primary cutaneous anaplastic large-cell lymphomas are ALK–,^121^ and ALK expression in the skin in the majority of skin lesions indicates systemic disease.^116^ However, as shown in a Workshop case of a 26-year-old male, rare cases of well-documented primary cutaneous anaplastic large-cell lymphoma are ALK+ (Figure 8).^122^,^123^ Conversely, it should also be remembered that ALK negativity in the skin does not exclude systemic disease, as 20–60% of anaplastic large-cell lymphomas can be ALK–.

Another Workshop case of primary cutaneous anaplastic large-cell lymphoma was ALK– but expressed EMA. Like ALK, EMA is more often present in systemic anaplastic large-cell lymphoma with secondary skin involvement rather than in primary cutaneous anaplastic large-cell lymphoma (67% versus 32%, respectively),^123^ so EMA expression cannot be used reliably to stage cutaneous anaplastic large-cell lymphoma. EMA may be expressed in large cells in 31% of lymphomatoid papulosis, making EMA of little use in distinguishing lymphomatoid papulosis Type C from primary cutaneous anaplastic large-cell lymphoma. If present, EMA would be useful in distinguishing anaplastic large-cell lymphoma from other reactive CD30+ cutaneous infiltrates (bug bites, drug reactions, viral infections, etc.). It should be mentioned that clusterin was initially thought to be exclusively expressed in systemic anaplastic large-cell lymphoma,^124^ but recent larger studies have documented clusterin expression in 41–100% of primary cutaneous anaplastic large-cell lymphomas.^125^–^127^ Careful staging and close clinical follow-up remain the most reliable methods to confirm a diagnosis of primary cutaneous disease.

Most studies have shown patients with systemic ALK+ anaplastic large-cell lymphoma have a favourable prognosis compared with the ALK– group.^98^,^99^,^117^,^118^ Patients with ALK+ tumours at extranodal sites, particularly the bone, may not always have a good prognosis.^128^ Other poor prognostic factors include small cell variant histology and peripheral blood involvement and CD56 expression.^118^,^129^–^131^ Primary CNS anaplastic large-cell lymphoma is very uncommon, being reported in less than 20 cases. ALK+ primary CNS anaplastic large-cell lymphomas (approximately 50%) have been reported to have a better prognosis.^132^ An unusual feature of primary CNS anaplastic large-cell lymphoma is the high degree of involvement of the dura or leptomeninges (69%).^131^,^132^ A CD56+, ALK+, t(2;5)(p23;q35)+, primary brain anaplastic large-cell lymphoma in a 27-year-old immunocompetent male was included in the Workshop (Figure 8). Despite the ALK+ phenotype, the patient had an aggressive course possibly related to CD56 expression by the tumour cells. As has been previously suggested in primary lymphoma of the peripheral nerve, CD56 (NCAM, neural cell adhesion molecule) expression may explain localization to the nervous system through homophilic binding of CD56+ tumour cells to CD56 on neural cells.^133^ It should be mentioned that ALK is weakly positive in normal glia, neurones and endothelial cells in the CNS^134^ but does not pose a problem in determining positivity in brain anaplastic large-cell lymphoma.^132^ ALK is expressed in neuroblastoma and glioblastoma, but these tumours are CD30–.

### How to classify alk–anaplastic large-cell lymphoma

Approximately 20–60% of anaplastic large-cell lymphomas are ALK–. Due to lack of a defined common pathogenic mechanism or specific clinicopathological features (other than primary cutaneous anaplastic large-cell lymphoma), haematopathologists have debated whether the diagnostic criteria for ALK– anaplastic large-cell lymphoma define a specific entity or if ALK– anaplastic large-cell lymphoma should be included in peripheral T-cell lymphoma unspecified. Three ALK–, CD30+ non-cutaneous T-cell lymphomas were included in the Workshop. Two were difficult to classify. One was a lung lesion with CD30 expression in a subset of the cells and an angiocentric growth pattern. The tumour cells were CD4+, CD5+ but lacked EMA, TIA-1, CD56 and EBV expression and the diagnosis was a CD30+ peripheral T-cell lymphoma unspecified. The second ALK– anaplastic large-cell lymphoma expressed CD15 and CD30 and the differential diagnosis was Hodgkin's lymphoma versus a CD15+ anaplastic large-cell lymphoma versus a CD15+, CD30+ peripheral T-cell lymphoma.^135^,^136^ Whether CD15 expression excludes a diagnosis of anaplastic large-cell lymphoma and whether CD15+, CD30+ T-cell lymphomas represent a specific entity will be answered when more cases have been studied.

The third ALK– anaplastic large-cell lymphoma case was a rare presentation of T-cell anaplastic large-cell lymphoma arising in the soft tissue around a breast implant in a 49-year-old female (Figure 8). Anaplastic large-cell lymphoma rarely presents in the breast as primary or secondary disease.^137^ If the previous reports, approximately 35% of anaplastic large-cell lymphomas in the breast have arisen in association with prosthetic implants^137^,^138^ and anaplastic large-cell lymphoma represents at least 50% of cases of lymphoma associated with breast implants.^137^ Other than an interesting hypothesis regarding the relationship of the prosthetic material to immune stimulation and the development of lymphoma, most of the discussion in this Workshop case centred around whether ALK– anaplastic large-cell lymphoma represents a distinct entity. The remainder of this section will discuss the case against placing ALK– anaplastic large-cell lymphoma back into the category peripheral T-cell lymphoma unspecified.

Comparative genomic hybridization studies comparing ALK– anaplastic large-cell lymphoma and peripheral T-cell lymphoma unspecified by Zettl *et al*. have shown important genetic differences. Although peripheral T-cell lymphoma unspecified and ALK– anaplastic large-cell lymphoma share recurrent chromosomal alterations (such as loss of 6q and 13q) frequently reported for other T- and B-cell non-Hodgkin lymphoma (NHL),^139^ the tumours show significant differences. Loss of 9p21-pter (31% in peripheral T-cell lymphoma unspecified and 0% in anaplastic large-cell lymphoma, Fisher's *P* = 0.0244) and 5q21 (33% in peripheral T-cell lymphoma unspecified versus 0% in anaplastic large-cell lymphoma, Fisher's *P* = 0.0126) is significantly more common in peripheral T-cell lymphoma unspecified. There is a trend for ALK– anaplastic large-cell lymphoma to have gains of chromosome 1q (46% versus 17%, Fisher's *P* = 0.0577). In addition, these data show a trend for both peripheral T-cell lymphoma unspecified and ALK- anaplastic large-cell lymphoma to have distinct genetic differences when compared with ETCL, T-prolymphocytic leukaemia and adult T-cell leukaemia/lymphoma. The degree of genetic instability varied between ALK– and ALK+ anaplastic large-cell lymphoma and peripheral T-cell lymphoma unspecified, with the greatest in the latter. Losses of 5q and 12q also appear to segregate peripheral T-cell lymphoma unspecified from other well-defined T-cell lymphomas.

A recent paper using FISH for the 2p23 locus showed extra copies of chromosome 2p23 in all five samples from four patients with ALK– anaplastic large-cell lymphoma and in five of seven primary cutaneous anaplastic large-cell lymphomas, but in none of six ALK+ anaplastic large-cell lymphomas.^140^ Zettl *et al*. found imbalances in chromosome 2 in five (26%) of nodal/systemic anaplastic large-cell lymphomas (one ALK+, four ALK–) and no primary cutaneous anaplastic large-cell lymphoma. Zettl *et al*. also reported solitary amplifications of 2cen-p22 or imbalances of chromosome 2 were present in 15/42 (31%) of peripheral T-cell lymphoma-NOS. Extra copies of chromosome 2 are not uncommon in lymphoma and have been reported in B- and T-cell lymphoma and Hodgkin's lymphoma in the Mitelman Database of Chromosome Aberrations in Cancer.^141^

A defect in T-cell receptor (TCR) αβ protein expression (based on immunohistochemical detection using the antibody βF-1) has been identified in > 90% of ALK+ and ALK– anaplastic large-cell lymphomas^142^ compared with loss of βF-1 expression in only approximately 10% of peripheral T-cell lymphoma unspecified and no angioimmunoblastic T-cell lymphomas (AILT). Ninety-six percent of ALK+ and 40% of ALK– anaplastic large-cell lymphomas also lacked expression of the CD3 antigen, compared with 29% of peripheral T-cell lymphomas NOS and 20% of AILT. CD3 molecules are associated with the TCR protein and transduce the signal of TCR engagement to ZAP-70, a tyrosine kinase that integrates cognate and costimulatory signals for downstream signalling. As further evidence of a defect in TCR signalling, ZAP-70 is lost in anaplastic large-cell lymphoma compared with other T-cell lymphomas.^143^ Overall, ZAP-70 is detected in 25–30% of anaplastic large-cell lymphomas (8–25% of ALK+ and 20–41% ALK–) versus 59–74% peripheral T-cell lymphomas NOS and 29–57% of primary cutaneous (PC) anaplastic large-cell lymphomas.^143^,^144^

Virtually all (82–100%) systemic anaplastic large-cell lymphomas, including ALK+ and ALK– cases, and 41–100% of PC anaplastic large-cell lymphomas express clusterin, a ubiquitous glycoprotein encoded by a gene on chromosome 8p21. Clusterin has many proposed functions including complement regulation, cell aggregation, lipid transport and response to cell injury or stress. Clusterin is expressed in a Golgi-associated pattern in anaplastic large-cell lymphoma.^125^–^127^ In contrast, clusterin is present in only 3.5% of peripheral T-cell lymphoma unspecified cases from three series^124^,^125^,^127^ and only 13% of other T-cell neoplasms were positive and usually showed a more diffuse cytoplasmic staining rather than the more distinct Golgi pattern of reactivity seen in anaplastic large-cell lymphoma.^125^,^127^

Recent microarray analysis of ALK+ and ALK– anaplastic large-cell lymphoma (including primary cutaneous anaplastic large-cell lymphoma) has shown ALK+ compared with ALK– anaplastic large-cell lymphoma over-expresses genes encoding signal transduction molecules (*SYK*, *LYN*, *CDC37*) and underexpresses transcription factor genes (including *HOXC6* and *HOX A3*).^145^ Both groups highly expressed kinase genes (*LCK*, protein kinase C, *vav2* and *NKIAMRE*) and anti-apoptotic molecules, suggesting overlap in pathogenesis; a comparison with peripheral T-cell lymphoma unspecified was not performed in this study.

In summary, since the original description there have been difficulties in diagnosis and classification of anaplastic large-cell lymphoma. Frequent involvement of extranodal sites, the promiscuity of CD30, expression of EMA with frequent lack of lymphocyte common antigen (LCA) and, as cases in this session pointed out, rare keratin and myeloid antigen expression contribute to the potential misdiagnosis. Recognition of the t(2;5) and abnormal expression of ALK in lymphoid tissue have further clarified our understanding of anaplastic large-cell lymphoma and helped in its diagnosis, particularly at extranodal sites or where other antigens may be expressed. ALK expression is usually associated with systemic disease and a better prognosis, but there are important exceptions. ALK expression can rarely be seen in anaplastic large-cell lymphoma limited to the skin; there is currently no substitute for careful staging and follow-up. In ALK+ anaplastic large-cell lymphoma other factors such as CD56 may indicate a poor prognosis.

Lastly, the classification of ALK– anaplastic large-cell lymphoma is controversial. Current genetic and biological features provide evidence that ALK– anaplastic large-cell lymphoma is more closely related to ALK+ anaplastic large-cell lymphoma than peripheral T-cell lymphoma unspecified and strongly support inclusion of ALK– CD30+ lymphomas with anaplastic large-cell lymphoma in future lymphoma classification. However, to make a diagnosis of ALK– anaplastic large-cell lymphoma there must be strict adherence to characteristic cytology (a large cell predominant population with abundant cytoplasm and pleomorphic, embryo or hallmark nuclei or wreath-like giant cells) and strong CD30 expression with a membrane and Golgi distribution in virtually every cell. In lymph nodes, some involvement of sinuses should be seen. Fortunately, most small cell predominant or lymphohistiocytic variants of anaplastic large-cell lymphoma are ALK+, as a diagnosis of ALK– anaplastic large-cell lymphoma with variant histology would be particularly difficult; large CD30+ hallmark type cells must be present with a preferential distribution of the CD30+ large cells around blood vessels.^131^ In ALK– anaplastic large-cell lymphoma with a null phenotype (and genotype), immunostains to exclude other tumours such as Hodgkin lymphoma (HL), carcinoma and acute leukaemia must be performed (Table 6).

**Table 6:** Anaplastic large-cell lymphoma and Its differential diagnosis, summary from the Workshop

ALK expression correlates with young age, systemic disease, a cytotoxic epithelial membrane antigen- positive phenotype and usually a good prognosis, but there are important exceptions to this rule
A significant number of anaplastic large-cell lymphoma cases (40–65%) have extranodal disease either at a primary site or as part of a systemic process. The determination of the origin of extranodal anaplastic large-cell lymphoma (primary versus a systemic process) is critically important, particularly in the skin
ALK expression alone is not diagnostic of anaplastic large- cell lymphoma as ALK can be present in other tumours, principally inflammatory myofibroblastic and other soft tissue tumours, tumours of neural origin (neuroblastoma, glioblastoma), and a very rare ALK+ B-cell lymphoma
To make a diagnosis of ALK-negative anaplastic large-cell lymph oma there must be strict adherence to characteristic cytology (a large cell predominant population with abundant cytoplasm and pleomorphic, embryo or hallmark nuclei or wreath-like giant cells) and strong CD30 expression with a membrane and Golgi distribution in virtually every cell. In lymph nodes, some involvement of sinuses should be seen

## ABBREVIATIONS

ALK: anaplastic lymphoma kinase
CTCL: cutaneous T-cell lymphoma
DLBCL: diffuse large B-cell lymphoma
ETCL: enteropathy-type T-cell lymphoma
FL: follicular lymphoma
HAART: highly active antiretroviral therapy
IDA-LPDs: immunodeficiency-associated lymphoproliferative disorders
IELs: intraepithelial lymphocytes
LEL: lymphoepithelial lesion
MALT: mucosa-associated lymphoid tissue
MM: multiple myeloma
MZBCL: marginal zone B-cell lymphomas
PCFCL: primary cutaneous follicle centre lymphoma
PCLBCL: primary cutaneous diffuse large B-cell lymphoma
PEL: primary effusion lymphoma
PID: primary immune disorders
PTLD: post-transplant lymphoproliferative disorder
SPLTCL: subcutaneous panniculitic-like T-cell lymphoma
